# The effect of interlaminar Coflex stabilization in the topping-off procedure on local and global spinal sagittal alignment

**DOI:** 10.1186/s12891-023-06231-1

**Published:** 2023-02-11

**Authors:** Dong-Fan Wang, Wei-Guo Zhu, Wei Wang, Chao Kong, Shi-Bao Lu

**Affiliations:** 1 Department of Orthopedics, Xuanwu Hospital, Capital Medical University, No.45 Changchun Street, Xicheng District, Beijing, 100053 China; 2 National Center for Clinical Research on Geriatric Diseases, No.45 Changchun Street, Xicheng District, Beijing, China

**Keywords:** Coflex, Topping-off procedure, Interlaminar dynamic stabilization, Sagittal spinal alignment, Degenerative lumbar spinal stenosis

## Abstract

**Purpose:**

To investigate the effect of interlaminar Coflex stabilization (ICS) at various segments in the topping-off procedure on local and global spinal sagittal alignment.

**Methods:**

Eighty-nine consecutive patients with degenerative lumbar spinal stenosis (DLSS) who underwent ICS and transforaminal lumbar interbody fusion (TLIF) were retrospectively reviewed. They were divided into Group A (L4-L5 ICS + L5-S1 TLIF), Group B (L3-L4 ICS + L4-S1 TLIF), and Group C (L2-L3 ICS + L3-S1 TLIF) according to their fusion levels. The measured local sagittal parameters included the implanted segmental angle (ISA), intervertebral disc angle (IDA), intervertebral foreman height (IFH), and disc height. The assessed global sagittal parameters included thoracic kyphosis, lumbar lordosis (LL), the fused segment angle (FSA), the sacral slope, the pelvic tilt, pelvic incidence, and the sagittal vertical axis. The Oswestry Disability Index (ODI) and visual analog scales (VAS) were recorded to evaluate the clinical outcomes.

**Results:**

Regarding the local alignment parameters, the ISA and IDA decreased immediately after surgery in Groups A and B, followed by an increase at the last follow-up (all, *P* < 0.05). Conversely, the IFH of Groups A and B first increased after surgery and then decreased to approximately the original value (all, *P* < 0.05). No significant differences were evident between the local sagittal parameters at different time points in Group C. Regarding the global sagittal profiles, the LL and FSA exhibited a significant postoperative increase (both at *P* < 0.05) in all the groups. All three groups displayed significant improvements in the ODI, VAS-back pain, and VAS-leg pain. Furthermore, 4.5% (4/89) of the patients exhibited radiographic adjacent segment degeneration (ASD) at the last follow-up.

**Conclusion:**

ICS during topping-off surgery led to a temporary loss of local lordosis, especially in the lower lumbar segment, while the intervertebral space realigned after middle-term follow-up. The topping-off procedure with ICS is a feasible and promising surgical option of DLSS since it reduces fusion levels and prevents ASD development.

## Introduction

Lumbar instrumentation and fusion is a conventional, classic surgical option for degenerative lumbar spinal stenosis (DLSS) [1]. Nevertheless, the fused segment leads to stress concentration at the adjacent level, which is associated with adjacent segment degeneration (ASD) development [2]. For the elderly population, especially, preventing ASD development has raised significant concern [3]. The high incidence of ASD, ranging from 21 to 75%, has facilitated the innovation and development of hybrid surgical techniques such as the ‘topping-off’ procedure, which consists of traditional fusion surgery and dynamic stabilized device implantation [4, 5].

The FDA-approved Coflex is a dynamic stabilized device commonly used in the topping-off procedure, allowing a smooth transition from caudal fused to cephalad motion-retained segments. Previous studies have reported that inserting Coflex can decelerate ASD by preserving segmental mobility and reducing the load of the intervertebral disk [6]. In a retrospective study involving 164 patients with DLSS, Chen et al. reported an ASD incidence of 13.2% in patients who underwent topping-off surgery after a four-year follow-up, while the fusion group displayed a percentage of 26.1% [7]. Yuan et al. studied 87 patients treated via Coflex interspinous stabilization or posterior lumbar interbody fusion (PLIF) and found that the ASD reoperation rate was significantly lower in the Coflex group than in the PLIF group (4.8% vs. 11.1%) [8]. Therefore, the Coflex device plays a role in improving clinical ASD progression.

Studies have shown that postoperative sagittal alignment correlates with long-term surgical outcomes [9]. Biomechanically, the Coflex insertion may result in the loss of lordosis of the implanted segment since this device is in contact with the sides of the cranial and caudal spinous processes and distracts the interspinous distance [10, 11]. To date, the influence of the Coflex technique in the topping-off procedure on local and global spinal sagittal alignment has not been clearly defined. Therefore, the purpose of this retrospective study was to analyze the variation in local and global sagittal alignment in patients who received a topping-off procedure with interlaminar Coflex stabilization (ICS) and further evaluate the effect of ICS on sagittal spinal alignment.

## Methods

### Patient population

After approval by the ethics committee at our hospital, a retrospective review was performed of 279 consecutive patients diagnosed with DLSS between January 2018 and August 2020. The inclusion requirements necessitated that patients were [7, 12] 1) aged 40–80 years and 2) underwent ICS and transforaminal lumbar interbody fusion (TLIF), 3) with a minimum follow-up of 12 months. The exclusion criteria included 1) previous spinal surgery, 2) ﻿osteopenia or osteoporosis, 3) degenerative lumbar scoliosis (Cobb angle > 25°), 4) cauda equina syndrome, 5) spinal infection, and 6) radiographically confirmed damage of the vertebrae caused by trauma or tumors. A total of 89 patients were included in the final analysis after selection.

Demographic data, such as age, sex, height, weight, and body mass index (BMI), were collected via electronic medical record reviews. The bone mineral density (BMD) at the lumbar spine was assessed via dual-energy X-ray absorptiometry.

The patients were classified into three groups based on the fusion levels. Patients with L5-S1 TLIF and ICS at L4-L5 were assigned to Group A, patients with L4-S1 TLIF and ICS at L3-L4 were assigned to Group B, and those with L3-S1 TLIF and ICS at L2-L3 were assigned to Group C.

### Surgical procedure

#### Transforaminal lumbar interbody fusion

Patients were placed prone after general anesthesia, and the surgical field was exposed through a midline longitudinal incision. Pedicle screws were sized and inserted bilaterally under C-arm X-ray guidance. Depending on the clinical presentation, a laminectomy, facetectomy, or both were performed on the more symptomatic side, followed by hypertrophic ligamentum flavum resection. This unilateral approach was used to perform a discectomy, after which the cartilaginous endplate was removed. Cancellous autograft was packed inside the intervertebral space, and a cage of the appropriate size was inserted. The rod-screw system was tightened after re-establishing the appropriate lordosis curvature.

#### Interlaminar Coflex insertion

**Fig. 1:**
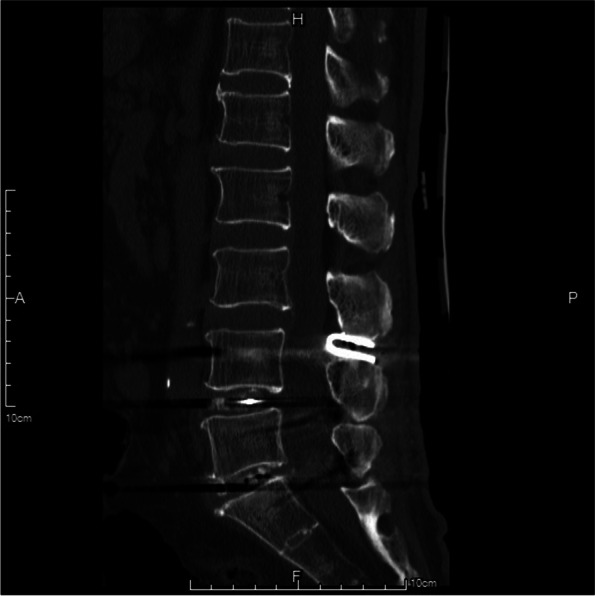
Schematic diagram of interlaminar Coflex insertion

Indications for ICS at the adjacent level of fusion segments were as follows: 1) radiological confirmation of at least moderate lumbar spinal stenosis (defined as > 25% reduction of the anteroposterior dimension compared with the normal level on CT/MRI) and 2) no segmental instability (defined as > 3 mm dynamic sagittal translation and > 10° dynamic angulation) [12, 13]. The interspinal ligament and ligamentum flavum of the superior adjacent segment were resected. Coflex of the appropriate size was introduced into the interlaminar space (Fig. 1). Anteroposterior and lateral view C-arm X-rays were performed to check the implant positions. The Coflex wings were tightened with a clamp after confirming the implantation depth.

Postoperative rehabilitation training was conducted as soon as possible. All patients were asked to wear a lumbar support belt for 12 weeks after surgery.

### Clinical assessment

The clinical outcomes were assessed via visual analog scale (VAS)-back pain, VAS-leg pain, and the Oswestry Disability Index (ODI) [14, 15]. VAS clinical efficacy was defined as a decrease > 2 points from the baseline. The ODI recovery rate was calculated as (postoperative ODI score - preoperative ODI score)/preoperative ODI score*100%. An improvement of more than 50% on the ODI from the baseline was deemed clinically effective. All clinical parameters were assessed and recorded by the same research assistant.

### Radiological evaluation

#### Local sagittal parameters

**Fig. 2:**
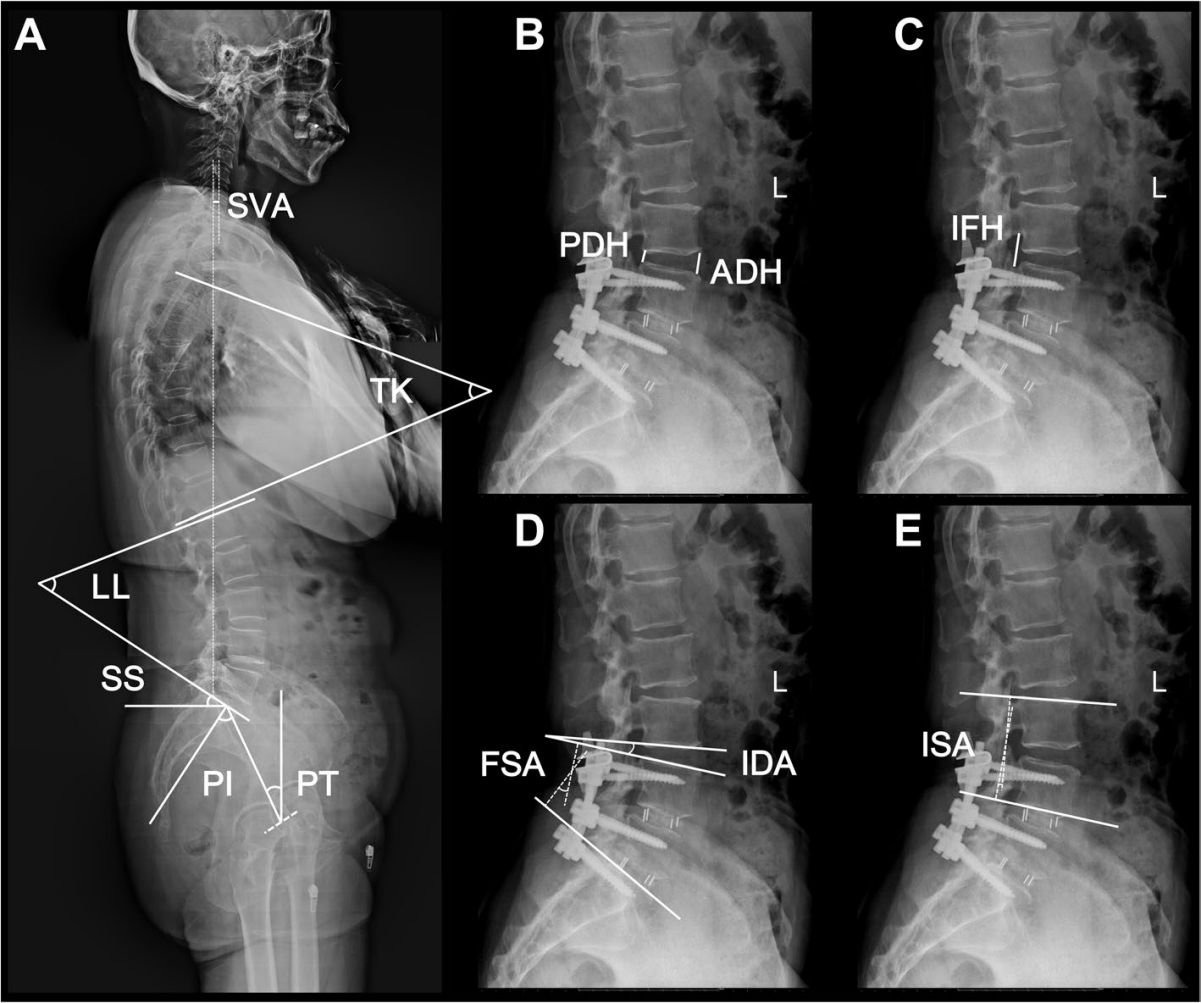
Radiographic evaluation of the spinal sagittal parameters. **A** SVA indicates sagittal vertical axis, TK indicates thoracic kyphosis, LL indicates lumbar lordosis, SS indicates sacral slope, PI indicates pelvic incidence, PT indicates pelvic tilt. **B** PDH indicates posterior disc height, ADH indicates anterior disc height. **C** IFH indicates intervertebral foramen height. **D** FSA indicates fused segment angle, IDA indicates intervertebral disc angle. **E** ISA indicates implanted segment angle

Pre- and postoperative whole-spine radiographs were obtained with the patients in an upright standing position in both anteroposterior and lateral views. The measured local radiological parameters included the following (Fig. 2): 1) The implanted segment angle (ISA): the Cobb angle between the superior endplate of the upper vertebra of the implanted segment and the inferior endplate of the lower vertebra of the implanted segment, 2) The intervertebral disc angle (IDA): the Cobb angle between the inferior endplate of the upper vertebra of the implanted segment and the superior endplate of the lower vertebra of the implanted segment. 3) The anterior disc height (ADH): the distance between the inferior anterior corner of the upper vertebral body and the superior anterior corner of the lower vertebral body. 4) The posterior disc height (PDH): the distance between the inferior posterior corner of the upper vertebral body and the superior posterior corner of the lower vertebral body. 5) The intervertebral foreman height (IFH): the maximum height between the inferior margin of the pedicle of the superior vertebra and the superior margin of the pedicle of the inferior vertebra.

The radiographic ASD criteria required at least one of the following parameters to be fulfilled: 1) Degenerative spondylolisthesis above grade I or a slip ≥4 mm [16]. 2) Segmental kyphosis > 10° [16]. 3) A disc height reduction ≥50% [17].

#### Global sagittal parameters

The measured global sagittal parameters included the following (Fig. 2): 1) Thoracic kyphosis (TK): the Cobb angle between the superior end plate of T4 and the inferior end plate of T12. 2) Lumbar lordosis (LL): the Cobb angle between the superior end plates of both L1 and S1. 3) Fused segment angle (FSA): the Cobb angle between the superior end plate of the upper vertebra of the fused segment and the inferior end plate of the lower vertebra of the fused segment. 4) The sacral slope (SS): the angle between the superior end plates of the sacrum and the horizontal line. 5) The pelvic tilt (PT): the angle between the line linking the midpoint of the superior end plate of S1 and the center of the femoral heads and vertical line. 6) Pelvic incidence (PI): the angle between the line linking the midpoint of the superior end plate of S1 and the center of the femoral heads and the line vertical to the superior end plate of the sacrum. 7) The sagittal vertical axis (SVA): the distance between the posterosuperior corner of S1 and the vertical line from the C7 body center. The lordosis distribution index (LDI) was calculated as ISA or FSA/LL*100%.

All the radiographic data measurements were conducted by two experienced spinal surgeons (Wang and Kong). The average of two measurements was regarded as the final result.

### Statistical analysis

All data were presented as the mean value ± standard deviation. The normal distribution was evaluated using the Shapiro-Wilk normality test. All the demographic and radiological data exhibited a normal distribution, while the clinical parameters did not. Continuous variables between the three groups were compared using one-way ANOVA and the Kruskal-Wallis test with Bonferroni or Tamhanes T2 post hoc analysis. The chi-square test was used to compare the gender composition ratios. The paired t-test and Wilcoxon matched-pairs signed rank tests were used to calculate the significance during pairwise comparisons. The intraclass coefficients (ICC) and 95% confidence intervals (CIs) for all the parameters were calculated to evaluate the intrinsic variability reliability of the radiographic measurements. ICC of all radiographic parameters was higher than 0.8.

The data were analyzed using SPSS Statistics (version 26.0, IBM Corp., Armonk, NY, USA), while the statistical significance was denoted by *P* < 0.05.

## Results

### The demographic characteristics and clinical parameters of the entire cohort

A total of 57 females and 32 males with a mean age of 65.96 ± 7.89 years participated in this study, while the average follow-up duration was 14.16 ± 3.01 months. Here, 26 patients underwent L4–5 Coflex and L5-S1 fusion, 49 were treated with L3–4 Coflex and L4-S1 fusion, and 14 received L2–3 Coflex and L3-S1 fusion treatment. The baseline demographic data of the three groups are summarized in Table 1. All the demographic parameters among the groups were approximated, including age, sex, height, weight, BMI, BMD, and follow-up duration (all, *P* > 0.05). Significant improvements from the baseline were observed in the VAS-Back pain, VAS-Leg pain, and ODI of all three groups (all, *P* < 0.001) (Table 2).

**Table 1:** Demographic characteristics of patients in three groups

	Group A (*n* = 26)	Group B (*n* = 49)	Group C (*n* = 14)	*P*
Age (years)	63.27 ± 10.24	67.14 ± 6.76	66.86 ± 5.92	0.118
Sex (Female/Male)	16:10	31:18	10:4	0.869
Height (cm)	163.38 ± 7.63	163.14 ± 7.17	163.29 ± 7.15	0.990
Weight (kg)	67.79 ± 9.25	68.27 ± 9.89	67.43 ± 10.54	0.953
BMI (kg/m^2^)	25.32 ± 2.46	25.67 ± 3.56	25.23 ± 3.00	0.851
Lumbar BMD (g/cm^2^)	1.17 ± 0.15	1.10 ± 0.15	1.16 ± 0.17	0.111
Follow-up periods (months)	13.96 ± 4.43	14.31 ± 1.90	14.00 ± 3.26	0.878

*BMI* body mass index, *BMD* bone mineral density

**Table 2:** Comparisons of clinical parameters before and after surgery

Variables		Group A (*n* = 26)	Group B (*n* = 49)	Group C (*n* = 14)	*P*
VAS-back pain (cm)	Pre-op	5.69 ± 1.01	5.73 ± 0.81	6.50 ± 0.85	0.012*
	Post-op	2.38 ± 0.64†	2.51 ± 0.82†	2.14 ± 0.86†	
	Last follow-up	1.96 ± 0.60†	2.39 ± 0.64†	1.92 ± 0.62†	
VAS-leg pain (cm)	Pre-op	6.62 ± 0.70	6.55 ± 1.10	7.57 ± 0.85	0.003**
	Post-op	2.42 ± 0.90†	2.65 ± 1.27†	3.29 ± 1.33†	
	Last follow-up	2.27 ± 0.60†	2.53 ± 0.94†	2.50 ± 0.76†	
ODI (%)	Pre-op	43.92 ± 7.84	46.61 ± 9.02	51.71 ± 9.14	0.030*
	Post-op	27.15 ± 5.13†	26.98 ± 5.37†	31.57 ± 6.80†	
	Last follow-up	22.69 ± 3.48†	24.41 ± 4.32†	26.71 ± 4.05†	

Group A indicates the group including patients who undergo L4–5 Coflex + L5-S1 fusion. Group B indicates the group including patients who undergo L3–4 Coflex + L4-S1 fusion. Group C indicates the group including patients who undergo L2–3 Coflex + L3-S1 fusion

*VAS* visual analog scale, *ODI* Oswestry Disability Index

†, *P* < 0.05 when post-op and last follow-up compared to pre-op

*, *P* < 0.05

**, *P* < 0.01

### The comparison of the local sagittal parameters at different time points

The variation in the local sagittal parameters is summarized in Table 3. Regarding the angle parameters, the ISA of Group A decreased from 12.60° ± 6.61° to 10.77° ± 5.57° (*P* = 0.007) after surgery, followed by an increase to 11.99° ± 5.68° (*P* = 0.030) at the last follow-up. Group B displayed an initial ISA decline from 6.22° ± 6.56° to 4.45° ± 5.89° (*P* < 0.001), followed by an increase to 5.80° ± 6.64° (*P* < 0.001). The postoperative IDA in Group A decreased from 10.92° ± 4.89° to 8.37° ± 4.28° (*P* = 0.001) and then increased to 10.80° ± 4.01° (*P* < 0.001) at the last follow-up. The IDA of Group B initially decreased from 6.89° ± 3.01° to 5.10° ± 2.60° (*P* < 0.001), followed by an increase to 6.80° ± 2.87° (*P* < 0.001). As to the distance parameters, the PDH of Group A displayed an initial increase from 6.88 mm to 7.91 mm (*P* < 0.001) and then decreased to 6.82 mm (*P* < 0.001) at the last follow-up. Although Groups A and B exhibited a 1 mm increase (both, *P* < 0.001) in the IFH immediately after surgery, the final IFH values were similar to those of the preoperative period in the two groups. No significant differences were evident between the local sagittal parameters in Group C at the various time points.

**Table 3:** Comparisons of local sagittal parameters before and after surgery

Variables		Coflex at L4–5 (*n* = 26)	Coflex at L3–4 (*n* = 49)	Coflex at L2–3 (*n* = 14)	*P*
ISA (°)	Pre-op	12.60 ± 6.61	6.22 ± 6.56	0.07 ± 6.27	0.000**
	Post-op	10.77 ± 5.57‡	4.45 ± 5.89‡	−0.81 ± 6.29	
	Last follow-up	11.99 ± 5.68	5.80 ± 6.64	0.10 ± 7.21	
IDA (°)	Pre-op	10.92 ± 4.89	6.89 ± 3.01	2.38 ± 1.65	0.000**
	Post-op	8.37 ± 4.28‡	5.10 ± 2.60‡	1.96 ± 1.61	
	Last follow-up	10.80 ± 4.01	6.80 ± 2.87	2.43 ± 2.56	
ADH (mm)	Pre-op	14.08 ± 3.58	11.26 ± 3.03	8.79 ± 2.77	0.000**
	Post-op	13.76 ± 3.46	11.31 ± 3.01	8.71 ± 2.88	
	Last follow-up	13.77 ± 3.32	10.88 ± 3.02	8.59 ± 2.88	
PDH (mm)	Pre-op	6.88 ± 1.90	6.18 ± 1.72	4.42 ± 1.21	0.000**
	Post-op	7.91 ± 2.43‡	6.65 ± 1.82	4.92 ± 1.22	
	Last follow-up	6.82 ± 2.16	5.94 ± 1.58§	4.11 ± 1.09§	
IFH (mm)	Pre-op	20.83 ± 2.59	20.64 ± 1.98	19.99 ± 1.95	0.502
	Post-op	21.78 ± 2.22‡	21.88 ± 1.89‡	20.24 ± 1.72	
	Last follow-up	20.68 ± 2.45	20.55 ± 1.98	19.59 ± 1.88	
Radiographic ASD (n)		2	1	1	NA

*ISA* implanted segment angle, *IDA* intervertebral disc angle, *ADH* anterior disc height, *PDH* posterior disc height, *IFH* intervertebral foramen height, *ASD* adjacent segment degeneration

‡, *P* < 0.05 when post-op compared to pre-op and last follow-up

§, *P* < 0.05 when last follow-up compared to post-op

**, *P* < 0.01

**Fig. 3:**
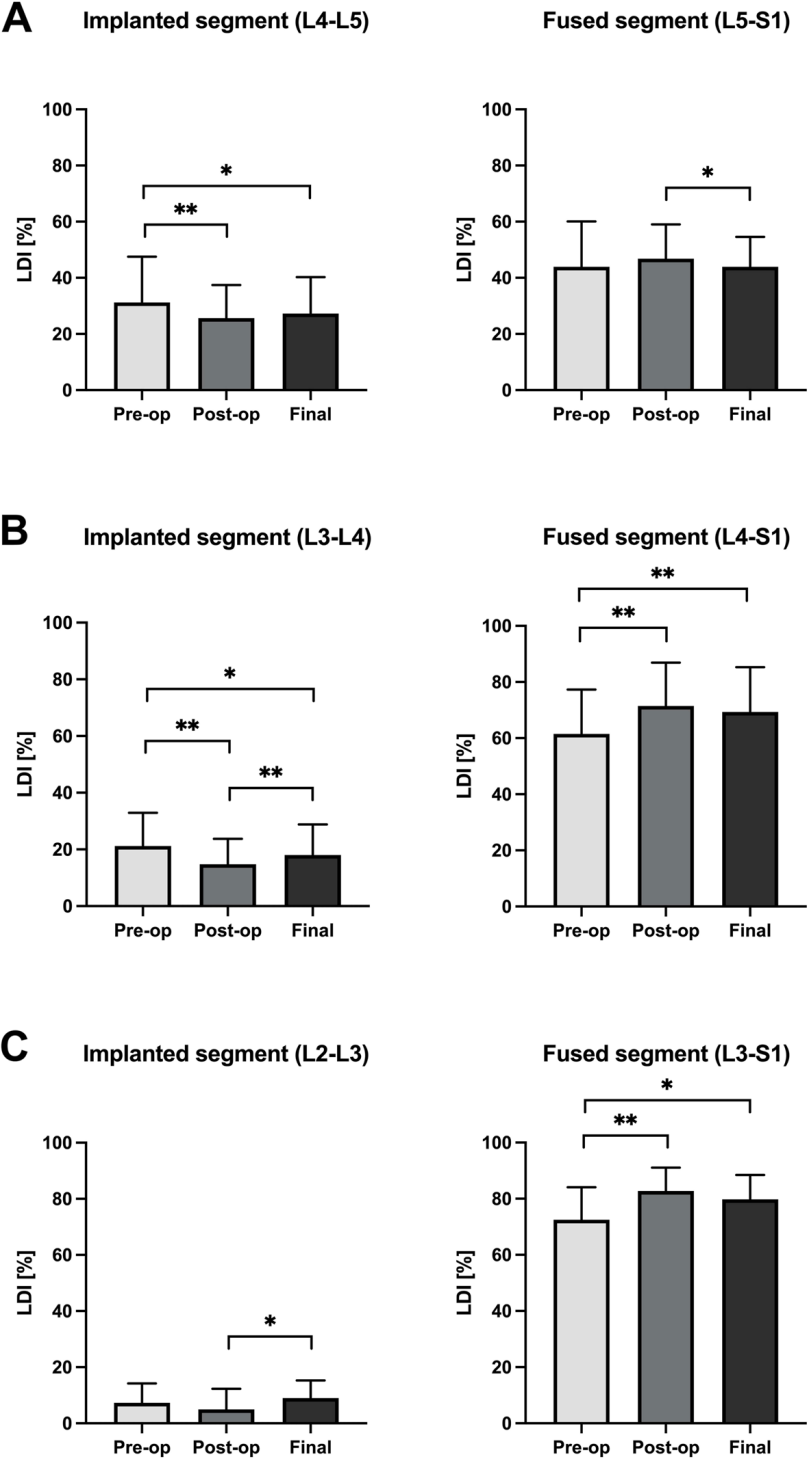
Illustration of the variations in lordosis distribution index (LDI) of the implanted or fused segment in three groups after surgery. **A** Variations in LDI of the implanted segment (left) and the fused segment (right) in Group A. **B** Variations in LDI of the implanted segment (left) and the fused segment (right) in Group B. **C** Variations in LDI of the implanted segment (left) and the fused segment (right) in Group C

The LDI variations are illustrated in Fig. 3. The LDI of the implanted segment decreased significantly (both, *P* < 0.01) after surgery in Groups A and B. Although the implanted segmental LDI values in these two groups increased slightly during the follow-up duration, they remained lower than in the preoperative period until the last follow-up (both, *P* < 0.05). In Group C, the LDI of the implanted segment was approximate among the different periods.

At the last follow-up, four patients exhibited signs of radiographic ASD (Table 3). Two Group A patients showed degenerative spondylolisthesis ≥4 mm and disc height reduction ≥50%, respectively. One patient exhibited disc height loss in Group B, while one presented segmental kyphosis > 10° in Group C.

### The evaluation of the global sagittal profiles at the various time points

Table 4 exhibits the variation in the global sagittal parameters. The LL of Group A increased from 41.70° ± 14.78° to 45.70° ± 9.62° after surgery (*P* = 0.015). In Group B, the postoperative LL increased from 37.80° ± 11.63° to 40.76° ± 10.35° (*P* = 0.002), while a 7° increase was evident in Group C after surgery (*P* = 0.009). Furthermore, postoperative FSA increased from 16.72° ± 4.60° to 19.30° ± 3.87° in Group A (*P* = 0.001), from 23.71° ± 7.94° to 28.14° ± 6.85° (*P* = 0.013) in Group B, and from 26.18° ± 10.74° to 32.29° ± 7.37° in Group C. The fused segmental LDI of Group B and C exhibited a notable increase (both, *P* < 0.01) after surgery (Fig. 3, B and C), with minimal variation during the follow-up period. Contrarily, this value was approximate in Group A at the various time points (Fig. 3, A).

**Table 4:** Comparisons of global sagittal parameters before and after surgery

Variables		Group A (*n* = 26)	Group B (*n* = 49)	Group C (*n* = 14)	*P*
TK (°)	Pre-op	−31.57 ± 9.62	−30.04 ± 11.84	−29.86 ± 11.60	0.833
	Post-op	−32.15 ± 7.60	29.81 ± 10.69	−29.91 ± 8.92	
	Last follow-up	−31.80 ± 8.18	−30.01 ± 9.85	−31.83 ± 9.40	
LL (°)	Pre-op	41.70 ± 14.78	37.80 ± 11.63	30.94 ± 13.14	0.046*
	Post-op	41.03 ± 18.20	39.96 ± 9.18†	36.27 ± 11.71†	
	Last follow-up	45.70 ± 9.62¶	40.76 ± 10.35†	37.10 ± 11.46†	
FSA (°)	Pre-op	16.72 ± 4.60	23.71 ± 7.94	26.18 ± 10.74	0.000**
	Post-op	19.58 ± 4.17†	28.50 ± 6.44†	32.61 ± 8.02†	
	Last follow-up	19.30 ± 3.87†	28.14 ± 6.85†	32.29 ± 7.37 †	
SS (°)	Pre-op	30.62 ± 7.08	28.11 ± 7.18	23.81 ± 6.35	0.017*
	Post-op	30.87 ± 6.53	29.30 ± 6.75	26.91 ± 7.79†	
	Last follow-up	31.15 ± 5.62	29.00 ± 6.82	26.39 ± 6.06†	
PT (°)	Pre-op	15.48 ± 7.33	18.48 ± 7.48	21.22 ± 6.67	0.040*
	Post-op	15.45 ± 7.75	17.39 ± 6.36	18.24 ± 6.67	
	Last follow-up	14.82 ± 6.93	17.83 ± 7.43	18.71 ± 7.19	
PI (°)	Pre-op	46.02 ± 9.73	46.38 ± 8.93	44.71 ± 7.88	0.829
	Post-op	46.24 ± 9.55	46.52 ± 8.91	44.80 ± 8.37	
	Last follow-up	45.91 ± 9.88	46.81 ± 9.30	45.06 ± 7.51	
SVA (cm)	Pre-op	0.78 ± 4.83	2.16 ± 4.31	3.03 ± 5.57	0.298
	Post-op	−0.24 ± 3.36	1.12 ± 2.99	1.69 ± 4.27	
	Last follow-up	−0.92 ± 2.01	1.05 ± 3.43	1.47 ± 3.26	

Group A indicates the group including patients who undergo L4–5 Coflex + L5-S1 fusion. Group B indicates the group including patients who undergo L3–4 Coflex + L4-S1 fusion. Group C indicates the group including patients who undergo L2–3 Coflex + L3-S1 fusion

*TK* thoracic kyphosis, *LL* lumbar lordosis, *FSA* fused segment angle, *SS* sacral slope, *PT* pelvic tilt, *PI* pelvic incidence, *SVA* sagittal vertical axis

¶, *P* < 0.05 when last follow-up compared to pre-op and post-op

†, *P* < 0.05 when post-op and last follow-up compared to pre-op

*, *P* < 0.05

**, *P* < 0.01

## Discussion

The advantages of the topping-off technique in treating DLSS and preventing ASD have been widely demonstrated by previous studies [7, 8, 18]. However, this device is prone to decreasing segmental local lordosis due to the implantation site and mechanical characteristics. To date, information regarding the variation in local or global sagittal alignment after ICS during the topping-off procedure is still lacking. The present study evaluated the variation in the sagittal spinal alignment and the presence of radiographic ASD in patients who underwent the topping-off procedure with ICS. The results indicated that the ICS technique had minimal adverse effects on local or global sagittal alignment and contributed to ASD prevention.

The efficacy of ICS involves increasing the IFH and decreasing the disk stress peak while simultaneously distracting the interspinous space, which may cause segmental lordosis loss [6]. In the present study, the ISA and IDA decreased significantly after surgery when the implanted segment was located at the lower lumbar region (L3-L4 or L4-L5), while these parameters were similar pre- and postoperatively in patients who received ICS in the upper lumbar region (L2-L3) (Table 3). The same ISA variation tendency was observed during the LDI analysis (Fig. 3). This phenomenon corresponds to the fact that the caudal part of the lumbar spine occupies a larger proportion of the global lordosis curvature. Roussouly et al. reported a lower LL arc with an average value of 39.9°, while the upper LL was only approximately 20° in the asymptomatic population [19]. Pan et al. presented a predictive formula (lower LL = 0.607*PI + 0.177 (*R*^2^ = 0.433)) that further substantiated the notion mentioned above [20]. In anatomical terms, the degree of segmental lordosis influences the interspinous distance since the rotational center in flexion-extension movements is located at the posterior portion of the intervertebral disk. The interspinous distance decreases with increased segmental lordosis. Therefore, the effect of Coflex device implantation on segmental lordosis depends on the selection of the implanted lumbar level. ICS at the caudal lumbar segment may result in a more pronounced local lordosis loss due to a lower interspinous distance in these segments. Conversely, a higher implanted segment corresponds to lower postoperative segmental lordosis loss.

From a structural perspective, the Coflex device functions as an elastic damping element to constrain the range of motion of the implanted spinal level [21]. During the movement of the ICS segment, the apex of the U-portion sustains the majority of the load, while the arms of the Coflex implant endure less stress but has some flexibility [6, 22]. Therefore, a slight deformation may occur in the U-shape arms of the Coflex with prolonged time after implantation, eventually leading to changes in the local sagittal parameters. This study found that the ISA and IDA increased while the IFH decreased from the postoperative period to the last follow-up, especially in patients who underwent ICS at L3-L4 and L4-L5. The final ISA, IDA, and IFH results were almost identical to the initial values (Table 3). This variation suggests that the lordosis loss mentioned before can be restored over time, which may be expected as an adaptive change by the implanted segment to maintain better local sagittal alignment. Similarly, Du et al. indicated that the distracting effect of the Coflex insertion was attenuated by follow-up duration extension in patients treated with dynamic stabilization only [23]. However, the LDI of the implanted segment in those patients was significantly lower at the last follow-up than during the preoperative period (Fig. 3). This result occurred because the LL, as the denominator in the LDI calculation, increased significantly after surgery (Table 4). Consequently, ICS did not negatively impact the overall maintenance of spinal lordosis.

**Fig. 4:**
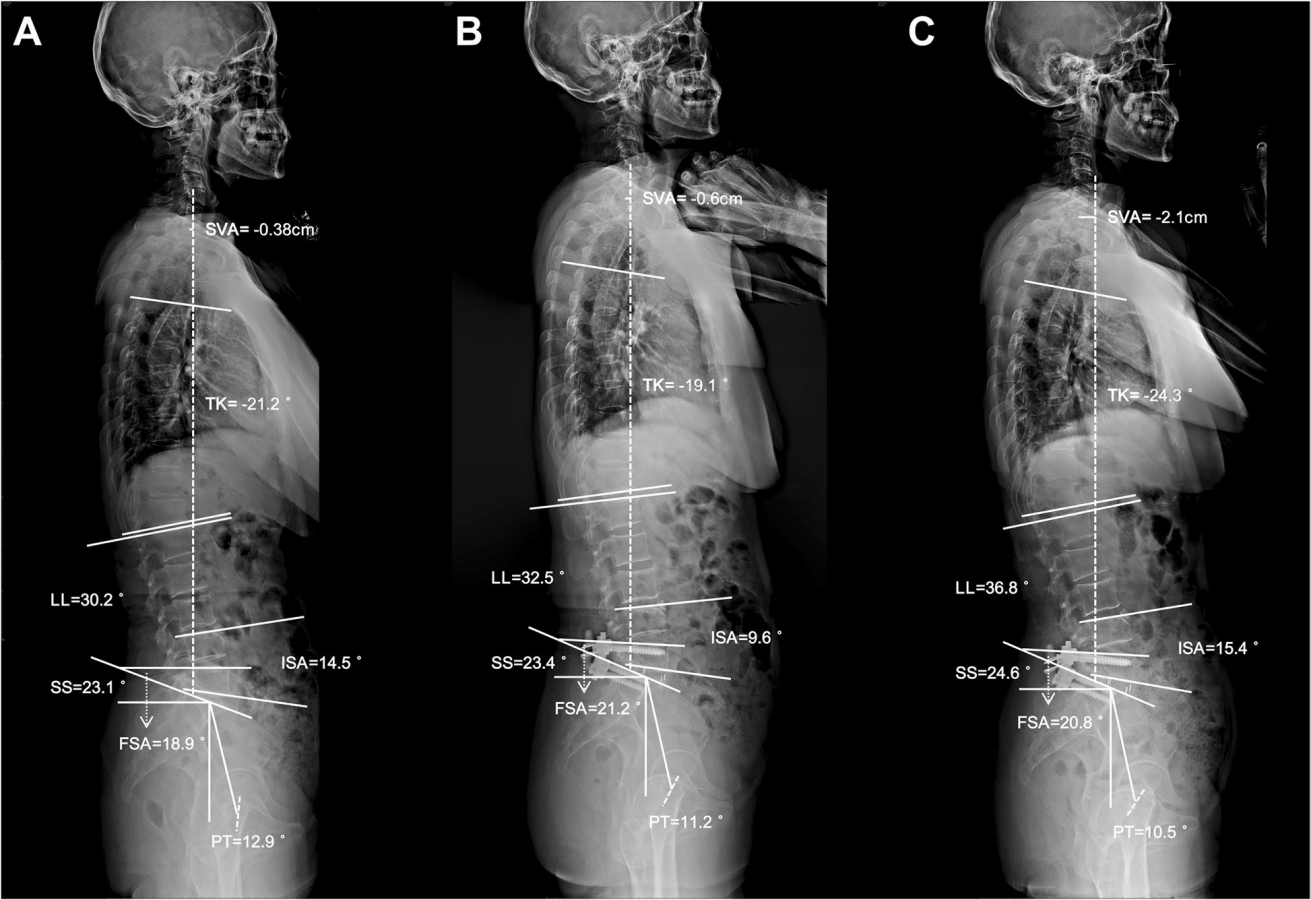
A 69-year-old female patient underwent L4-L5 ICS and L5-S1 TLIF with a 15-month follow-up. **A** Preoperative ISA, FSA, and LL was 14.5°, 18.9°, and 30.2°, respectively. **B** ISA decreased from 14.5° to 9.6° after surgery, while postoperative FSA and LL increased to 21.2° and 32.5°, respectively. **C** At the last follow-up, the ISA, FSA, and LL was 15.4°, 20.8°, and 36.8°, respectively

**Fig. 5:**
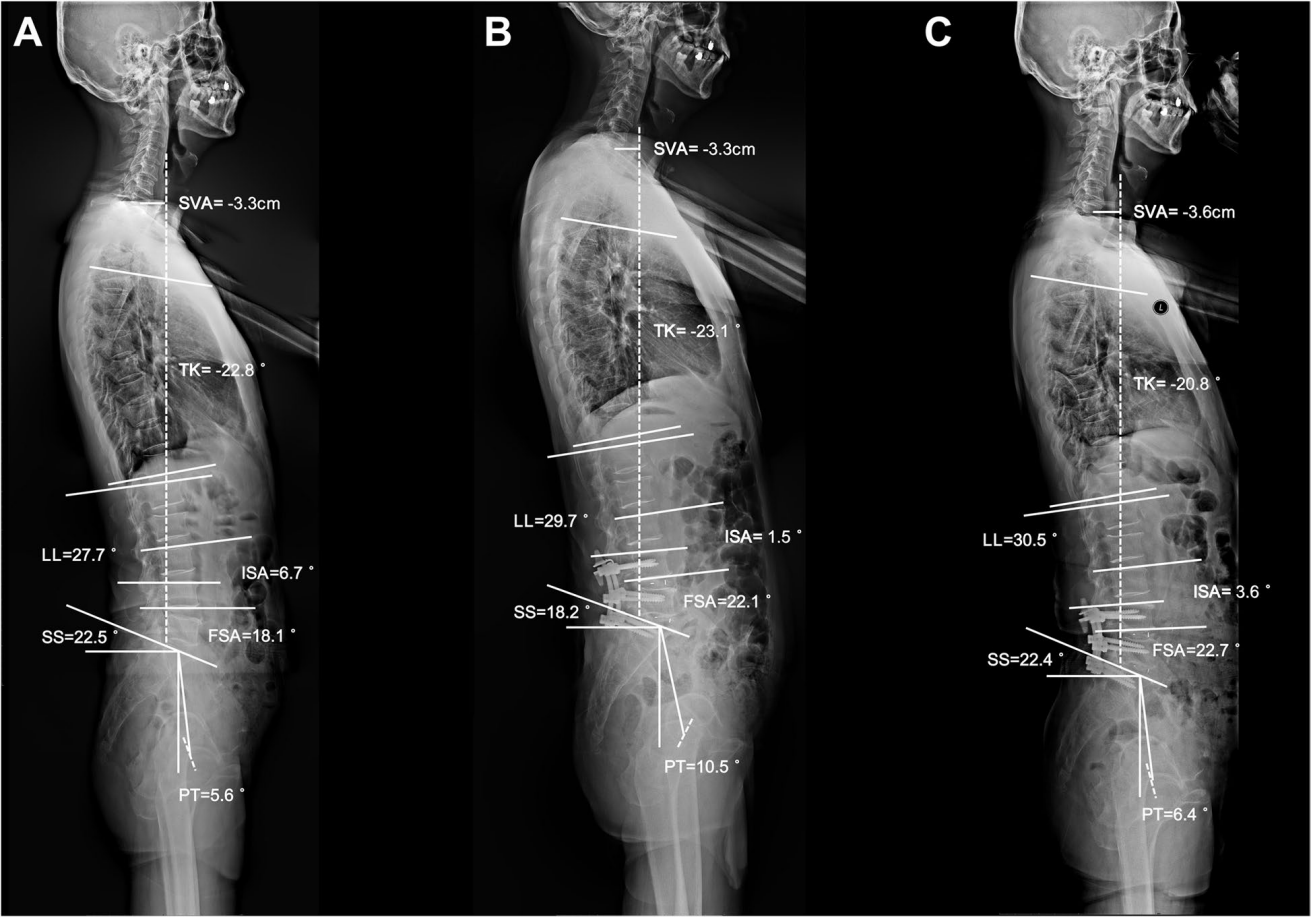
A 68-year-old male patient underwent L3-L4 ICS and L4-S1 TLIF with a 16-month follow-up. **A** Preoperative ISA, FSA, and LL was 6.7°, 18.1°, and 27.7°, respectively. **B** ISA decreased from 6.7° to 1.5° after surgery, while postoperative FSA and LL increased to 22.1° and 29.7°, respectively. **C** At the last follow-up, the ISA, FSA, and LL was 3.6°, 22.7°, and 30.5°, respectively

**Fig. 6:**
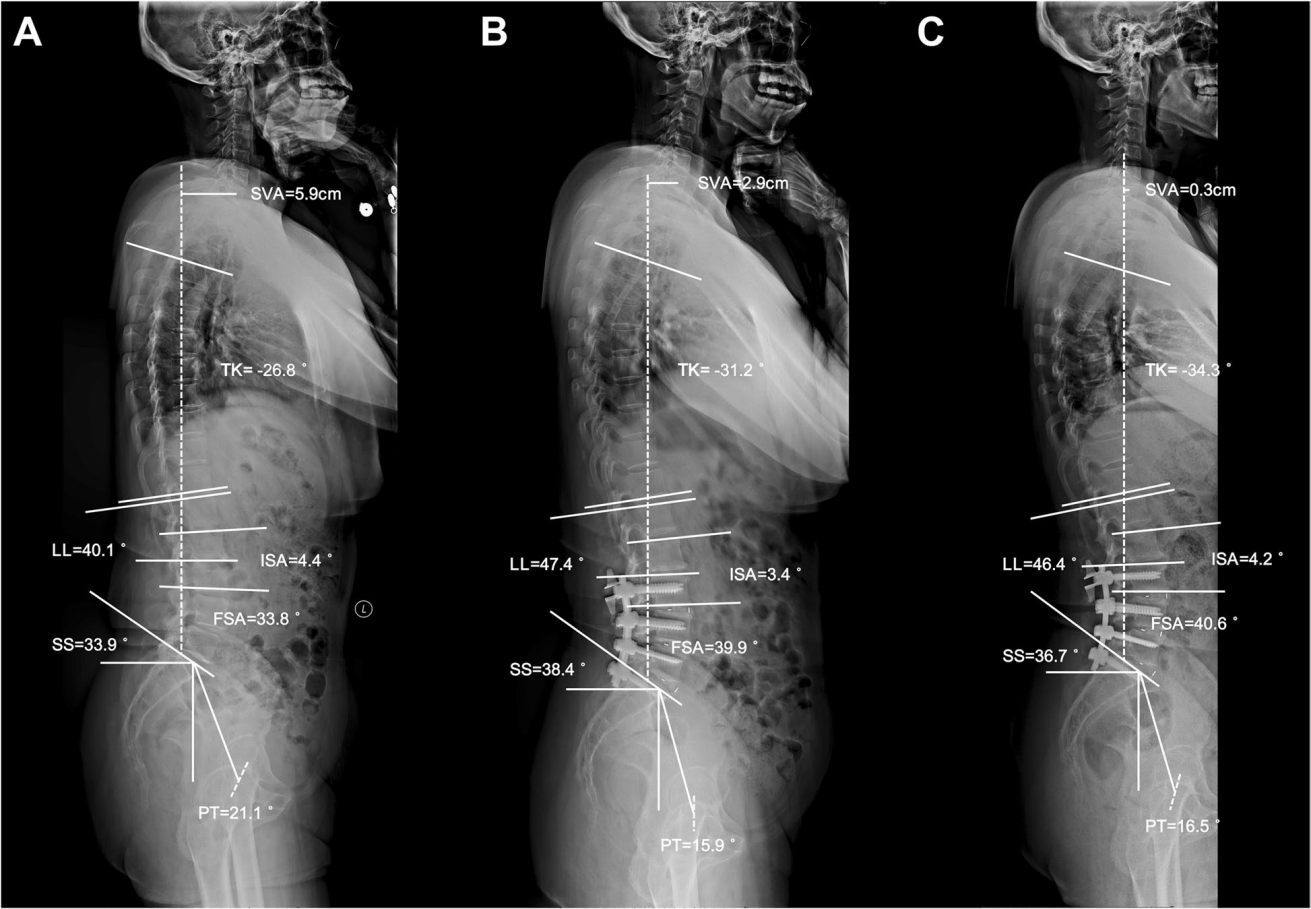
A 73-year-old female patient underwent L2-L3 ICS and L3-S1 TLIF with a 15-month follow-up. **A** Preoperative ISA, FSA, and LL was 4.4°, 33.8°, and 40.1°, respectively. **B** Postoperative ISA, FSA, and LL was 3.4°, 39.9°, and 47.4°, respectively. **C** At the last follow-up, the ISA, FSA, and LL was 4.2°, 40.6°, and 46.4°, respectively

In addition, global sagittal alignment correction has become an important goal since these parameters are correlated with clinical outcomes [24]. In this study, the global sagittal profiles, and clinical outcome indicators improved significantly at each postoperative time point (Tables 2 and 4). Lumbar fusion adjacent to the ICS played a major role, as the fused segments accounted for most of the lordosis distribution (Fig. 3). In a retrospective case-control study containing 88 patients who received one- or two-level lumbar fusion surgery, Cho et al. reported that most patients showed a significant improvement in postoperative LL, VAS-back pain, VAS-leg pain, and ODI [25]. Overall, patients with DLSS can benefit from hybrid surgery involving ICS and fusion in terms of sagittal spinal alignment and clinical efficacy. Moreover, the interlaminar Coflex insertion does not deteriorate segmental lordosis or the global sagittal profiles from a long-term perspective. The clinical cases of the three groups are shown in Figs. 4, 5 and 6.

For elderly patients with symptomatic DLSS, decompression and fusion surgery for the responsible levels have been recognized as important means for improving the prognosis [26]. Nevertheless, the fusion procedure is excessive regarding the adjacent lumbar segments with stenosis but no related symptoms, though these segments are more prone to ASD after surgery. The topping-off procedure can effectively prevent spinal stenosis deterioration at the adjacent level by resecting the thickening ligamentum flavum and stabilize adjacent segments via ICS to reduce ASD incidence. As reported by previous studies, the radiographic ASD incidence ranged from 4.4 to 24% after the topping-off procedure, depending on the definition of ASD, follow-up duration, and demographic characteristics of the patients [27, 28]. In this study, 4.5% (4/89) of the patients exhibited radiographic ASD at the last follow-up, while none required revision surgery due to symptomatic ASD. No device-related complications were found up to the last follow-up.

This study presented several limitations. First, potential biases were unavoidable because of the retrospective nature of this study. Radiographic parameter acquisition standardization was attempted by conducting comprehensive training before the study and illustrating each measurement in the statistical tables. Second, the sample size and follow-up duration were limited. Research with a larger cohort and longer follow-up duration is still ongoing to investigate the long-term effect of ICS on sagittal spinal alignments and clinical outcomes. Regardless of these drawbacks, this study highlights the impact of ICS after lumbar fusion on local and global sagittal spinal alignment and broadens the understanding of the topping-off technique.

## Conclusions

Interlaminar Coflex insertion during topping-off surgery leads to a temporary loss of implanted segment lordosis, especially in the lower lumbar segment and the intervertebral space realigned after middle-term follow-up. The results indicate that this technique minimally influences local and global spinal sagittal alignment. The topping-off procedure with ICS is a feasible and promising surgical option of DLSS since it reduces fusion levels and prevents ASD development.

## Data Availability

The datasets used and/or analyzed during the current study available from the corresponding author on reasonable request.
